# Anti-Inflammatory Effects of the Algal Diterpenoid Ruguloptone A by Modulation of M2 Response in Early Diabetic Retinopathy

**DOI:** 10.3390/pharmaceutics18050606

**Published:** 2026-05-15

**Authors:** Belén Cuevas, Eva Zubía, Francisco Martín-Loro, Ana I. Arroba

**Affiliations:** 1 Instituto de Investigación e Innovación en Ciencias Biomédicas de la Provincia de Cádiz (INiBICA), Hospital Universitario Puerta del Mar, 11009 Cádiz, Spain; belen.cuevagomez@alum.uca.es (B.C.); francisco.martin@inibica.es (F.M.-L.); 2 Departamento de Química Orgánica, Facultad de Ciencias del Mar y Ambientales, Universidad de Cádiz, 11510 Puerto Real, Spain; 3 Departamento de Endocrinología y Nutrición, Hospital Universitario Puerta del Mar, 11009 Cádiz, Spain

**Keywords:** diabetic retinopathy, M2 response, algal diterpenoids, inflammation

## Abstract

**Background/Objectives:** Inflammation is a critical contributor to the development of diabetic retinopathy (DR). In the early stages of DR, the compromised permeability of the blood–retina barrier facilitates the infiltration of macrophages and the activation of microglia. These specific retinal immune cells can adopt morphologies M1 or M2, linked to pro- or anti-inflammatory responses, respectively. This dual response represents a new therapeutic target against DR progression. This study aimed to investigate the modulation of the response M1/M2 and the molecular mechanism of two algal diterpenoids, rugukadiol A (RK) and ruguloptone A (RL), in the early inflammatory events associated with DR. **Methods:** LPS-stimulated microglial (Bv.2) and macrophage (RAW264.7) cells and an ex vivo physiological model of DR were used to analyze the effects of RK and RL on M1 and M2 inflammatory markers. **Results:** Compounds RK and RL, besides decreasing the expression of the M1 pro-inflammatory factors iNOS, *Il6* mRNA, and NLRP3 in LPS-stimulated Bv.2 cells, caused enhancements in *Arg-1* mRNA and *Il10* mRNA expression consistent with the induction of an M2 anti-inflammatory response. RK promoted p38α-MAPK phosphorylation, suggesting a non-classical activation of p38α related to the induction of anti-inflammatory responses. Consistently, treatment of retinal explants of BB rats in the early stages of DR with RL decreased M1 pro-inflammatory mediators and induced M2 anti-inflammatory markers, with a reduction in gliosis and a phenotype switch from activated to resting microglia. **Conclusions:** This study provides the first evidence of algal diterpenoids attenuating pro-inflammatory mediators and promoting the resolution of inflammation in a diabetic retinopathy context, thus opening the way to further explore this class of marine natural products and analogs for early DR management.

## 1. Introduction

The involvement of inflammation in metabolic disorders such as diabetes mellitus (DM) has extensively been studied in recent years, supporting the consideration of DM as a systemic, chronic, low-grade inflammatory disease [1]. Within DM, type 1 diabetes mellitus (T1DM) is an autoimmune disease that affects specific organs, such as the pancreas, with a later shift towards systemic compromise due to comorbidity and complications [2]. Among these, diabetic retinopathy (DR), which causes visual impairment and even blindness, is the most common complication [3], with an estimated prevalence of about 70% in T1DM patients after more than two decades of disease [4,5]. With an increasing number of diabetic patients and limited options for treatment, new therapeutic approaches are needed to improve the prevention and management of DR [6].

The innate immunity plays a critical role in diverse pathological conditions during the progression of T1DM and the development of DR as a specific complication [7]. A key feature of DR is the disruption of the blood–retina barrier (BRB) [8], which is associated with the inflammatory events occurring during DR progression [3]. Upon breakdown, the increased permeability of the BRB contributes to the recruitment of circulating immune cells to the site of damage, activating microglial cells and promoting the secretion of inflammatory mediators [9]. Macrophages, as systemic immune cells, and microglia, as immune cells in the central nervous system, are heterogeneous and plastic cells that display different morphologies and surface molecules in response to different exogenous danger signals [10,11]. In a classical activation, also known as M1 or pro-inflammatory response, both types of immune cells experience physiological changes and initiate signal transduction cascades that lead to abnormal production of chemokines, cytokines, and toxic mediators, which can further enhance inflammation and contribute to autoimmune pathologies such as T1DM and its complications [9,12]. In particular, the M1 phenotype of immune cells expresses nitric oxide synthase (iNOS), whose activation promotes the synthesis of different inflammatory mediators and facilitates the inflammatory response [13]. Activated microglial and macrophage cells are also able to generate an M2 or anti-inflammatory response that contributes to the resolution, tissue repair or defense of inflammation by producing anti-inflammatory molecules and tissue remodeling growth factors such as IL-10 and transforming growth factor beta (TGF-β), among others [10]. Moreover, the M2 phenotype of immune cells modulates arginine metabolism, thus regulating cell proliferation and tissue repair, and inhibiting inflammation [14]. During DR, activated macrophage and microglial cells give rise to the expression of both pro- and anti-inflammatory mediators [15]. Moreover, the dynamics of microglia switching between M1/M2 status have been shown to contribute to the differential immune response that occurs during DR progression [16].

On the other hand, the retina has antioxidant sensors that protect against the oxidative component of the diabetic milieu. In this regard, we have previously shown the increase in hemeoxygenase-1 (HO-1) expression during the early stages of DR [17,18,19]. HO-1, encoded by the HMOX1 gene in humans or the *Hmox1* gene in rodents, is an antioxidant enzyme that represents an important adaptive mechanism of various tissues, including the retinal tissue, for moderating the severity of cell damage produced by oxidative stress [20]. Moreover, the induction of HO-1 gene expression is mediated, among others, by p38α MAPK [21]. Interestingly, the increased expression of HO-1 modulates the inhibition of iNOS, a key enzyme of the M1 pro-inflammatory response of immune cells, and also induces arginase-1 expression, an enzyme associated with the M2 anti-inflammatory response [22,23].

**Figure 1:**
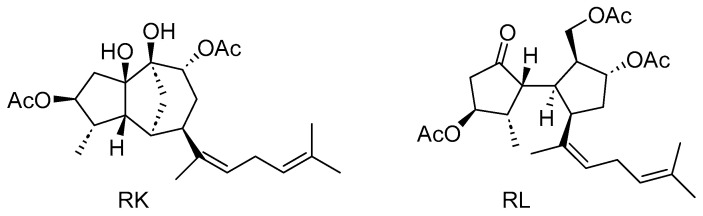
Chemical structures of rugukadiol A (RK) and ruguloptone A (RL).

Therefore, molecules that target macrophage/microglia polarization by modulating the switch from M1 to M2 may be promising for the treatment of the inflammation associated with DR. In this regard, a variety of plant-derived compounds with anti-inflammatory properties have been reported to act as modulators of M1/M2 phenotypes of macrophages [24]. Macroalgae, also known as seaweeds, are a rich source of bioactive natural products (specialized metabolites), most of which are of the terpenoid class [25], whose anti-inflammatory properties remain underexplored [26]. Recently, we have isolated from the brown alga *Rugulopteryx okamurae* a variety of diterpenoids, which have exhibited anti-inflammatory effects in LPS-stimulated Bv.2 and RAW 264.7 cells [27,28]. Among the tested compounds, rugukadiol A (RK) and ruguloptone A (RL) (Figure 1) caused significant inhibition of NO production and of *Nos2* and *Il1b* expression [27]. These findings, together with the lack of anti-inflammatory studies on any other compound belonging to these structural classes, prompted us to obtain further insights into the activity of the diterpenoids RK and RL.

This study aimed to evaluate the anti-inflammatory effects of RK and RL in Bv.2 microglial cells and RAW 264.7 macrophages as well as in retinal explants of BB rats in the context of inflammation and oxidative stress associated with early DR. Compounds RK and RL were found to enhance markers of M2 polarization in microglial but not in macrophage cells. The compound RL exhibited the best anti-inflammatory effects, which involved a non-classical activation of p38α MAPK, inducing a significant M2 anti-inflammatory response in microglial cells and in retinal explants of BB rats used as a model of DR, with an enhancement in *Hmox1* expression, *Il10*, and *Arg1*.

## 2. Materials and Methods

### 2.1. General Experimental Materials and Reagents

Fetal bovine serum (FBS) and culture media were supplied from Invitrogen (Grand Island, NY, USA). Bovine serum albumin (BSA), Triton X-100, sucrose, dimethylsulfoxide (DMSO), lipopolysaccharide (LPS), sodium dodecyl sulfate (SDS), penicillin/streptomycin and DL-Dithiothreitol (DTT) were obtained from Sigma-Aldrich (St. Louis, MO, USA). EDTA-free complete protease inhibitor cocktail was purchased from Roche (Basel, Switzerland). Accutase solution was acquired from BioLegend (San Diego, CA, USA). Acrylamide reagents and PVDF membranes for immunoblotting were purchased from Bio-Rad (Madrid, Spain). Protein quantification was performed using the BCA assay kit from Thermo Fisher (Waltham, MA, USA). Fluoromount-G mounting medium was obtained from Southern Biotech (Birmingham, AL, USA). Thiobarbital (0.5 g) was supplied by Braun Medical, S.A. (Rubí, Barcelona, Spain), whereas L-glutamine was acquired from Gibco (Carlsbad, CA, USA). The compounds RK and RL (purity ≥ 97%) were isolated from the brown macroalga *Rugulopteryx okamurae* and structurally characterized by spectroscopic analyses as previously described [27].

### 2.2. Antibodies

Primary antibodies against p65-NFkB (p65) and phospho-p38α MAPK (Thr180/Tyr182), and anti-p38α MAPK antibodies were purchased from Cell Signaling Technology (Danvers, MA, USA). Antibodies recognizing iNOS and HO-1 were obtained from Abcam (Cambridge, UK). The anti-arginase-1 antibody was supplied by BD Bioscience (Madrid, Spain). The anti-GFAP antibody was acquired from DAKO (Glostrup, Denmark), whereas the anti-α-tubulin antibody was purchased from Sigma-Aldrich (St. Louis, MO, USA). The IBA-1 antibody was obtained from Fujifilm-Wako Chemicals (Richmond, VA, USA). The antibody against NLRP3 was purchased from AdipoGene Life Sciences (Liestal, Switzerland). Horseradish peroxidase-conjugated secondary antibodies against mouse or rabbit IgG were acquired from Sigma-Aldrich (St. Louis, MO, USA). DAPI (4,6-diamidino-2-phenylindole) was supplied by ThermoFisher Scientific (Waltham, MA, USA).

### 2.3. Cell Culture

Bv.2 microglial cells were obtained from ACCEGEN Biotechnology (Farfield, CT, USA). The RAW 264.7 macrophage cell line was kindly provided by Dr. Valverde (IIBm “Alberto Sols”, UAM-CSIC, Madrid, Spain). Cells were seeded in a 6-well plate (Sarstedt, Nümbrecht, Germany) at a density of 1.5 × 10^5^ cells/well. Cultures were maintained at 37 °C in a humidified incubator containing 5% CO_2_ using RPMI medium supplemented with 10% heat-inactivated FBS, 1% penicillin/streptomycin, and 2 mM L-glutamine. Once cultures reached approximately 80% confluence, cells were rinsed twice with PBS and detached using Accutase reagent. Bv.2 and RAW 264.7 cells were pre-incubated with RK or RL (10 µM) for 3 h prior to stimulation with LPS (200 ng/mL) for an additional 24 h. The selected concentration of both compounds was established based on preliminary dose-response experiments. Under these conditions, neither RK nor RL altered cell viability, as previously described [27].

### 2.4. Animals

All procedures involving animals were approved by the Committee for the Ethical Use and Care of Experimental Animals (University of Cádiz, Spain) protocol number 005_abr20_PI2-ITI-012-2019. Animal handling and experimental procedures were conducted according FELASA recommendations and the guidelines established by the Association for Research in Vision and Ophthalmology (ARVO). Wild-type (WT) and bio-breeding (BB) rats were housed under controlled environmental conditions (20–21 °C, 12 h light/dark cycle) with unrestricted access to food and water, and blood glucose levels were monitored weekly in BB rats using tail-vein blood samples and a Freestyle Optium Neo (Abbott, Madrid, Spain). Animals were considered diabetics when glucose concentration exceeded 270 mg/dL (14.98 mmol/L). For ex vivo studies, retinas from 7-week-old male or female BB rats were collected after euthanasia by anesthesia overdose. The eyes were rapidly enucleated, and the anterior segment, lens, vitreous humor, retinal pigment epithelium and sclera were carefully removed. Retinal tissues were immediately frozen at –80 °C until RNA extraction.

### 2.5. Retinal Explants

Ex vivo retinal cultures were prepared from WT and BB rats aged 7 weeks. Retinas isolated from BB animals were maintained in serum-free R16 media (provided by Dr. P.A. Ekstrom, Lund University, Sweden). Explants were incubated in the absence or presence of RL (20 µM). The concentration selected for RL treatment was determined from preliminary dose-response analyses.

### 2.6. Immunofluorescence

Bv.2 microglial cells were plated on glass coverslips 24 h before treatment. Following stimulation and/or exposure to RL under serum-free conditions, cells were washed with PBS and fixed in 4% paraformaldehyde for 10 min at room temperature. After fixation, the cells were permeabilized with 0.4% Triton X-100 in PBS for 20 min and blocked for 2 h in PBS containing 3% BSA and 0.1% Triton X-100. The samples were incubated overnight at 4 °C with rabbit anti-p65 NFkB antibody (1:500) in blocking buffer. After washing, the cells were incubated for 2 h at room temperature in darkness with Alexa 488-conjugated anti-rabbit secondary antibody (1:1000; ThermoFisher Scientific, Waltham, MA, USA). For retinal explants, tissues were fixed in 4% paraformaldehyde for 24 h at 4 °C. Explants were subsequently washed in TBS containing 0.1% BSA and 0.1% Triton X-100, and permeabilized/blocked using TBS supplemented with 3% BSA and 1% Triton X-100 for 2 h. Retinal tissues were incubated overnight at 4 °C with anti-GFAP and anti-IBA-1 antibodies (1:500 each). Following washing steps, samples were incubated with Alexa Fluor 488-conjugated secondary antibody (1:1000, Molecular Probes, ThermoFisher Scientific, Waltham, MA, USA). Nuclei were counterstained with DAPI and samples were mounted using Fluoromount-G. Fluorescence images were acquired using a ZEISS inverted laser-scanning confocal microscope (ZEISS, Jena, Germany). For morphometric analyses, five images from independent retinal explants were captured with a 20X objective. IBA-1-positive microglial cells were quantified with ImageJ 1.54g software and categorized according to their morphology as either ramified or amoeboid. Ramified cells were defined by the presence of a small soma with at least three cellular projections, whereas amoeboid cells displayed an enlarged cell body with absent or minimal processes. All image analyses were performed by an investigator blinded to the experimental conditions.

### 2.7. Quantitative Real-Time Polymerase Chain Reaction (qPCR) Analysis

Total RNA was isolated using TRIzol^®^ reagent (Invitrogen, Madrid, Spain). Complementary DNA synthesis was performed with an iScript™ gDNA clear cDNA Synthesis kit (BioRad, Hercules, CA, USA) according to the manufacturer’s instructions. Quantitative PCR analyses were carried out using a CFX96 Touch Real-Time PCR Detection System (Biorad). Primers specific for mouse and/or rat for *Tnfa*, *Il6*, *Il1b*, *Il10*, *Nos2*, *Arg1*, *Hmox1* and *Actb* (Table S1) were purchased from Sigma-Aldrich (St. Louis, MO, USA).

### 2.8. Western Blotting

Protein extracts (20 µg) were separated by SDS-PAGE under denaturing conditions and electrotransferred onto PVDF membranes. The membranes were blocked using 5% nonfat dried milk or 3% BSA prepared in TBS (10 mM Tris-HCl, 150 mM NaCl, pH 7.5) and subsequently incubated overnight at 4 °C with the corresponding primary antibodies (generally diluted 1:1000) in TBS containing 0.05% Tween-20. After washing, membranes were incubated for 2 h at room temperature with HRP-conjugated secondary antibodies (1:2000 dilution). Immunoreactive bands were visualized using the Western-Bright Sirius chemiluminescent substrate from Advansta Inc (San José, CA, USA) and ChemiDoc^TM^ Imaging System (Bio-Rad).

### 2.9. Statistical Analysis

Densitometric analysis of immunoblots was performed using the ImageJ software. Data are expressed as means ± SEM. Statistical analyses were conducted using GraphPad Prism 7.0a (GraphPad Software, Boston, MA, USA). Differences among multiple groups were evaluated by one-way ANOVA followed by Bonferroni’s post hoc test, whereas comparisons between two groups were analyzed using Student’s paired *t*-test. Statistical significance was established at *p* < 0.05.

## 3. Results

### 3.1. Compounds RK and RL Decrease iNOS Protein and Il6 mRNA Levels in LPS-Stimulated Immune Cells

**Figure 2:**
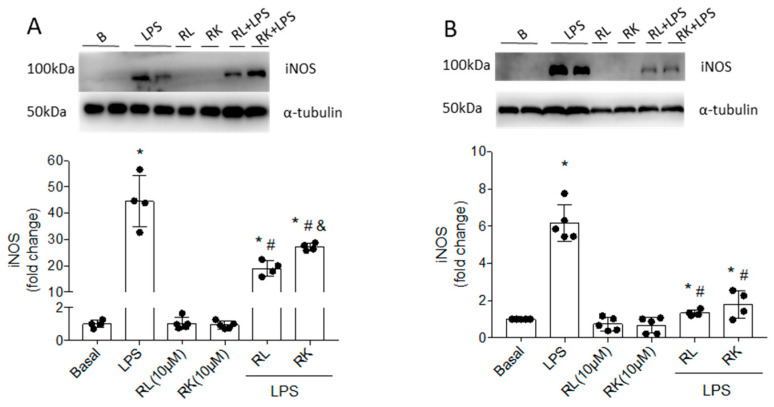
Inhibition of iNOS levels induced by compounds RK and RL in LPS-stimulated Bv.2 and RAW 264.7 cells. Cell cultures were treated with LPS (200 ng/mL), RK or RL (10 μM), LPS plus RK, or LPS plus RL for 24 h. iNOS protein levels were analyzed by Western blot in protein extracts from (**A**) Bv.2 microglial cells and (**B**) RAW 264.7 macrophage cells. α-Tubulin was used as a loading control. Representative blots are shown. The results are presented as means ± SEM (n = 5 independent experiments). The fold change relative to the basal condition is shown. * *p* ≤ 0.05 vs. basal, ^#^ *p* ≤ 0.05 vs. LPS, ^&^ *p* ≤ 0.05 vs. LPS plus RL treatment (one-way ANOVA followed by Bonferroni *t*-test).

In previous studies, we showed the non-cytotoxic effects and the potent inhibition of NO production caused by compounds RK and RL in microglial and macrophage cells, which are innate immune cells responsible for inflammatory response in DR [27]. In this study, we further supported the NO modulation caused by RK and RL by measuring the expression of iNOS protein levels when both immune cells were cultured with these compounds in the presence or absence of LPS stimulus. A low-dose LPS stimulation was used to induce a pro-inflammatory response that mimics the low-grade inflammation of a diabetic environment [17]. As shown in Figure 2A (Bv.2 cells) and Figure 2B (RAW 264.7 cells), treatment with RK and RL caused a significant reduction in the LPS-induced expression of iNOS protein levels compared to LPS-stimulated and non-treated cells. In both immune cell lines, the inhibition of iNOS protein levels was stronger in the treatment with RL (Table 1).

**Figure 3:**
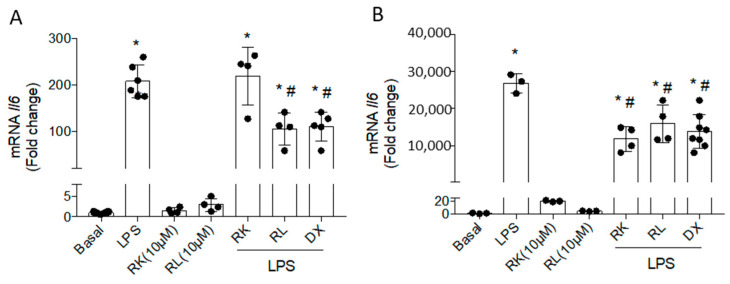
Inhibition of mRNA *Il6* caused by compounds RK and RL in LPS-stimulated Bv.2 and RAW 264.7 cells. Cell cultures were treated with LPS (200 ng/mL), RK or RL (10 μM), LPS plus RK, or LPS plus RL, and dexamethasone (DX) for 24 h. *Il6* mRNA values were determined by qRT-PCR in (**A**) Bv.2 microglial cells and (**B**) RAW 264.7 macrophage cells. Data were normalized to *Actb* gene expression. The results are mean ± SEM (n = 5 independent experiments). Fold changes are calculated relative to the basal value. * *p* ≤ 0.05 vs. basal, ^#^ *p* ≤ 0.05 vs. LPS (one-way ANOVA followed by Bonferroni *t*-test).

LPS stimulation of immune cells (macrophage and microglia) induces a classical activation or M1 pro-inflammatory response, promoting the synthesis and secretion of cytokines such as interleukins IL-1β and IL-6, and TNF-α. The compounds RK and RL have previously been observed to significantly reduce *Il1b* mRNA in LPS-stimulated Bv.2 microglial and RAW 264.7 macrophage cells, while *Tnfa* mRNA was not ameliorated [27]. In this study we analyzed IL-6, a cytokine involved in the central biological processes of inflammation and widely considered a marker in the context of diabetes, since it correlates with the risk of developing the disease [29]. As depicted in Figure 3A (Bv.2 cells) and Figure 3B (RAW 264.7 cells), the significant increases in *Il6* mRNA induced by LPS stimulation were decreased by treatment with RL (Table 1), in a similar extent to decreases caused by dexamethasone (DX) in each cell line.

**Figure 4:**
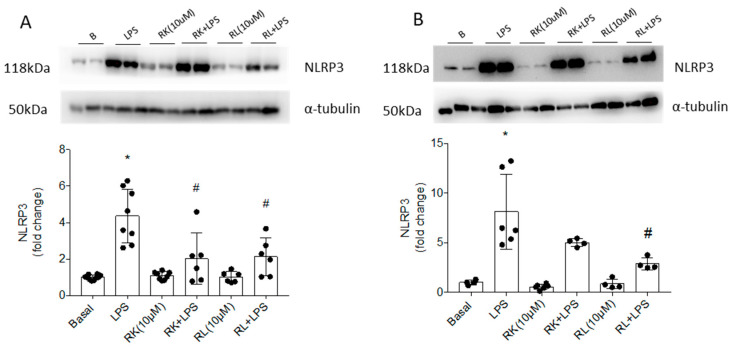
Protective effects of compounds RK and RL against LPS-mediated activation of the inflammasome in Bv.2 and RAW 264.7 cells. Cell cultures were treated with LPS (200 ng/mL), RK or RL (10 μM), LPS plus RK, or LPS plus RL for 24 h. Protein extracts from (**A**) Bv.2 microglial cells and (**B**) RAW 264.7 macrophage cells were analyzed by Western blot with antibody against NLRP3. α-Tubulin was used as a loading control. Representative blots are shown (n = 4–8 independent experiments). Blots were quantified by performing scanning densitometry. The results are mean ± SEM. The fold change relative to the basal condition is shown. * *p* ≤ 0.05 vs. basal, ^#^ *p* ≤ 0.05 vs. LPS (one-way ANOVA followed by Bonferroni *t*-test).

We next analyzed whether the anti-inflammatory effects of RK and RL were dependent of the inflammasome activation, since in LPS-stimulated immune cells IL1β is processed via caspase-1 through the NACHT, LRR and PYD domain-containing protein 3 (NLRP3) of the inflammasome complex [30]. The protein levels of NLRP3 were markedly increased in the LPS-stimulated condition in both immune cell lines, with a significant and similar reduction upon treatment with RK or RL in Bv.2 cells (Figure 4A). Both compounds also decreased NLRP3 protein levels in LPS-stimulated RAW 264.7 cells, although only the effect of RL was significant (Figure 4B) (Table 1).

### 3.2. Compounds RK and RL Induce an M2 Response Mediated by Arginase-1, Il10 and HO-1 in LPS-Stimulated Microglial Cells

**Figure 5:**
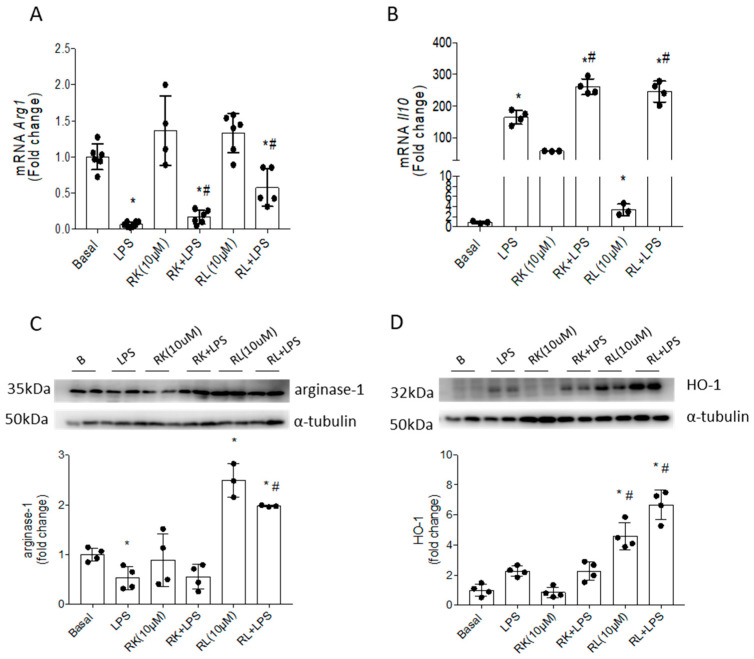
Induction of M2 response caused by compounds RK and RL in LPS-stimulated Bv.2 cells. Bv.2 microglial cells were treated with LPS (200 ng/mL), RK or RL (10 μM), LPS plus RK, or LPS plus RL for 24 h. (**A**) *Arg1* mRNA levels and (**B**) *Il10* mRNA levels were determined by qRT-PCR. Data were normalized to *Actb* gene expression. The results are mean ± SEM (n = 5 independent experiments). Protein extracts were analyzed by Western blot with antibodies against (**C**) arginase-1 and (**D**) HO-1. α-Tubulin was used as a loading control. Representative blots are shown (n = 4 independent experiments). Blots were quantified by performing scanning densitometry. The results are mean ± SEM. The fold change relative to the basal condition is shown. * *p* ≤ 0.05 vs. basal, ^#^ *p* ≤ 0.05 vs. LPS, treatment (one-way ANOVA followed by Bonferroni *t*-test).

In order to evaluate the capability of compounds RK and RL to induce anti-inflammatory responses, we studied arginase-1 expression (*Arg1 gene encode*), a marker of the M2 polarization stage of microglial cells, and *Il10* mRNA, an anti-inflammatory cytokine related to arginase-1 regulation. As shown in Figure 5A,B, LPS stimulation induced a significant reduction in *Arg1* mRNA levels and an increase in *Il10* mRNA levels in Bv.2 cells, which are changes associated with the early response to an insult.

The treatment of LPS-stimulated cells with RK or RL significantly reduced the effect of LPS on the decrease in *Arg1* mRNA. In addition, treatment with RK or RL led to *Il10* mRNA levels significantly higher than those caused by LPS alone. These results were consistent with the induction of an M2 anti-inflammatory response. Nonetheless, when the levels of the protein arginase-1 were analyzed (Figure 5C), only RL caused significant effects, increasing the levels of the protein in cells both alone and in the presence of LPS stimulation. Therefore, compound RL was identified to have potent effects on the induction of M2 response mediated by arginase-1.

It has been reported that HO-1 induction promotes the downregulation of pro-inflammatory cytokines, exerting protective effects (antioxidant and anti-inflammatory) in diabetic retina [31]. In this context, we analyzed the ability of compounds RK and RL to modify the expression levels of the protein HO-1 in Bv.2 cells. As shown in Figure 5D, only RL had a significant effect and increased HO-1 protein levels in cells both alone and in the presence of LPS.

On the other hand, RAW 264.7 cells treated either with compound RK or with compound RL showed a poor or negligible induction of M2 response (Figure S1).

### 3.3. Treatment with Compound RL Promotes Anti-Inflammatory Effects by Non-Canonical p38α-MAPK Activation

**Figure 6:**
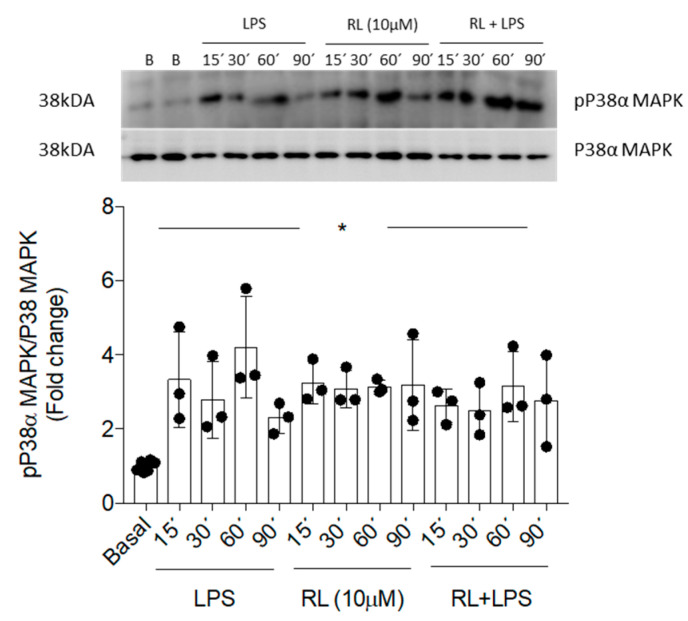
Effects of compound RL in the activation of p38α-MAPK in LPS-stimulated Bv.2 cells. Bv.2 microglial cells were treated with LPS (200 ng/mL), RL (10 μM), and LPS plus RL for the indicated periods of time. Protein extracts were analyzed by Western blot with antibodies against phosphorylated (p)-p38α-MAPK and total p38α-MAPK. Representative autoradiograms are shown (n = 3 independent experiments). Blots were quantified by performing scanning densitometry, and the results are mean ± SEM. The fold change relative to the basal condition is shown. * *p* ≤ 0.05 vs. basal (two-way ANOVA followed by Bonferroni *t*-test).

**Figure 7:**
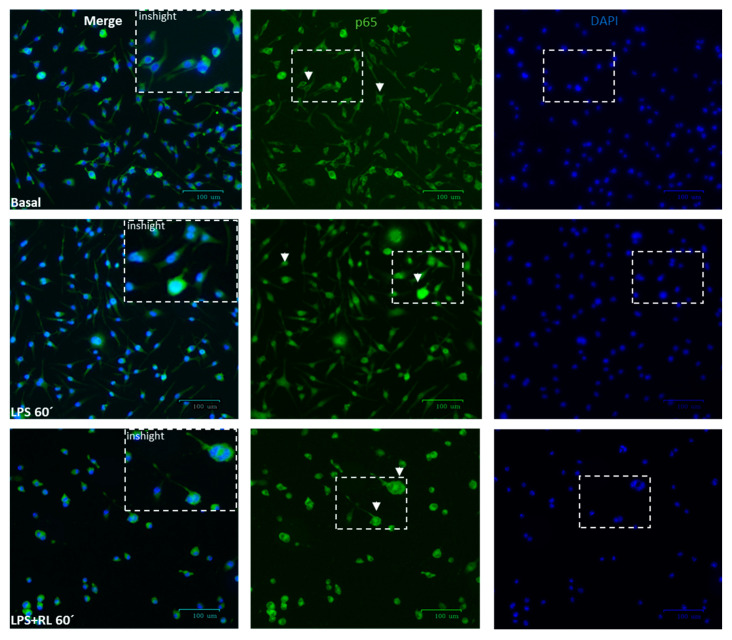
Effects of compound RL on the translocation of p65-NFκB in LPS-stimulated Bv.2 cells. Bv.2 microglial cells were treated with LPS (200 ng/mL), RL (10 μM), and LPS plus RL for the indicated periods of time. The nuclear translocation of p65-NF*κ*B was assessed by confocal immunofluorescence. Activation of p65-NF*κ*B and nuclear translocation was defined by an increase in immunofluorescence of p65-NF*κ*B (green channel) in the nuclear regions. Nuclear regions of Bv.2 microglial cells were determined by counterstaining nuclear DNA with DAPI (blue channel). White arrows indicate the p65-NF*κ*B nuclear or cytoplasmic localization. Insights in the merge column are magnifications of the areas marked with a dashed box in p65 and DAPI columns. Scale bar = 20 μm.

The previous results show the strong and consistent effects of RL as a pro-inflammatory inhibitor and anti-inflammatory promoter in microglial cell responses. Based on these results, we delved into the molecular mechanisms of RL by examining the p38α-MAPK- and NFκB-mediated signaling pathways in Bv.2 cells (Figure 6 and Figure 7).

It is well known that the phosphorylation of p38α-MAPK, a key protein of the MAPK signaling pathway, is critical for the ability of immune cells to respond to various pro-inflammatory stimuli, including LPS [32]. As shown in Figure 6, LPS stimulation induced rapid phosphorylation of p38α-MAPK, with the maximal effect being elicited after 60 min. Treatment with LPS and compound RL also led to significantly higher levels of phosphorylation of p38α-MAPK compared with the basal condition and similar to those induced by LPS alone. Moreover, treatment of microglial cells only with RL also caused a significant increase in the levels of phosphorylated p38α-MAPK, similar to those caused by LPS stimulus. However, the LPS-mediated nuclear translocation of p65-NFκB was prevented by pre-treatment with RL (Figure 7).

### 3.4. Compound RL Reduces the Early Inflammatory Response and Promotes the M2 Response in Retinal Explants from BB Rats

**Figure 8:**
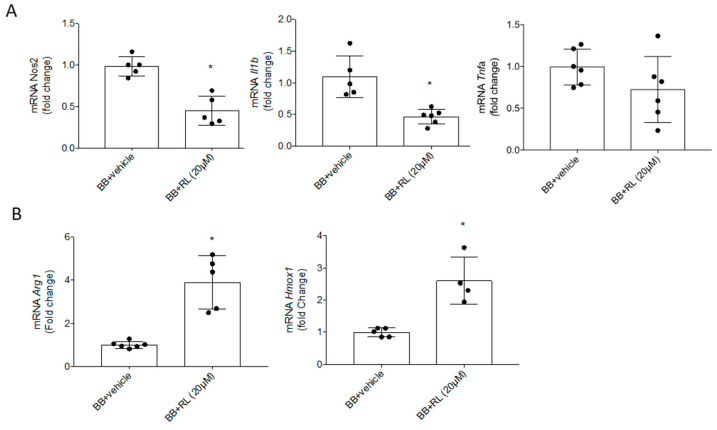
Ex vivo treatment with RL reduces neuroinflammation in BB rats during DR. Retinal explants from 7-week-old BB rats were treated with RL (20 μM) for 24 h. (**A**) *Nos2*, *Il1b* and *Tnfa* and (**B**) *Arg1* and *Hmox1* mRNA levels were determined by qRT-PCR. Data were normalized to *Actb* gene expression. The results are presented as mean ± SEM (n = 4–6 retinas per condition). Fold changes are calculated relative to the basal value. * *p* ≤ 0.05 vs. basal condition (*t*-test).

In order to obtain insights into the beneficial effects of compound RL on early inflammation associated with DR progression, we assayed RL on whole retinal explants from BB rats, an animal model for T1DM that shows early pro-inflammatory events linked to DR at 7 weeks old [19] (Figure S2). After treating retinal explants from BB rats for 24 h with RL (20 µM), the analysis of pro-inflammatory cytokines showed a reduction in mRNA levels of *Nos2* and *Il1b* compared to BB rats treated only with vehicle (Figure 8A), while *Arg1* and *Hmox1* mRNA levels, as anti-inflammatory mediators, were significantly increased (Figure 8B). These results indicate that RL decreases M1 or pro-inflammatory response and induces M2 or anti-inflammatory response in the retinas of BB rats.

### 3.5. Retinal Gliosis and Microglia Activation Are Modulated by the Treatment of Retinal Explants from BB Rats with Compound RL

**Figure 9:**
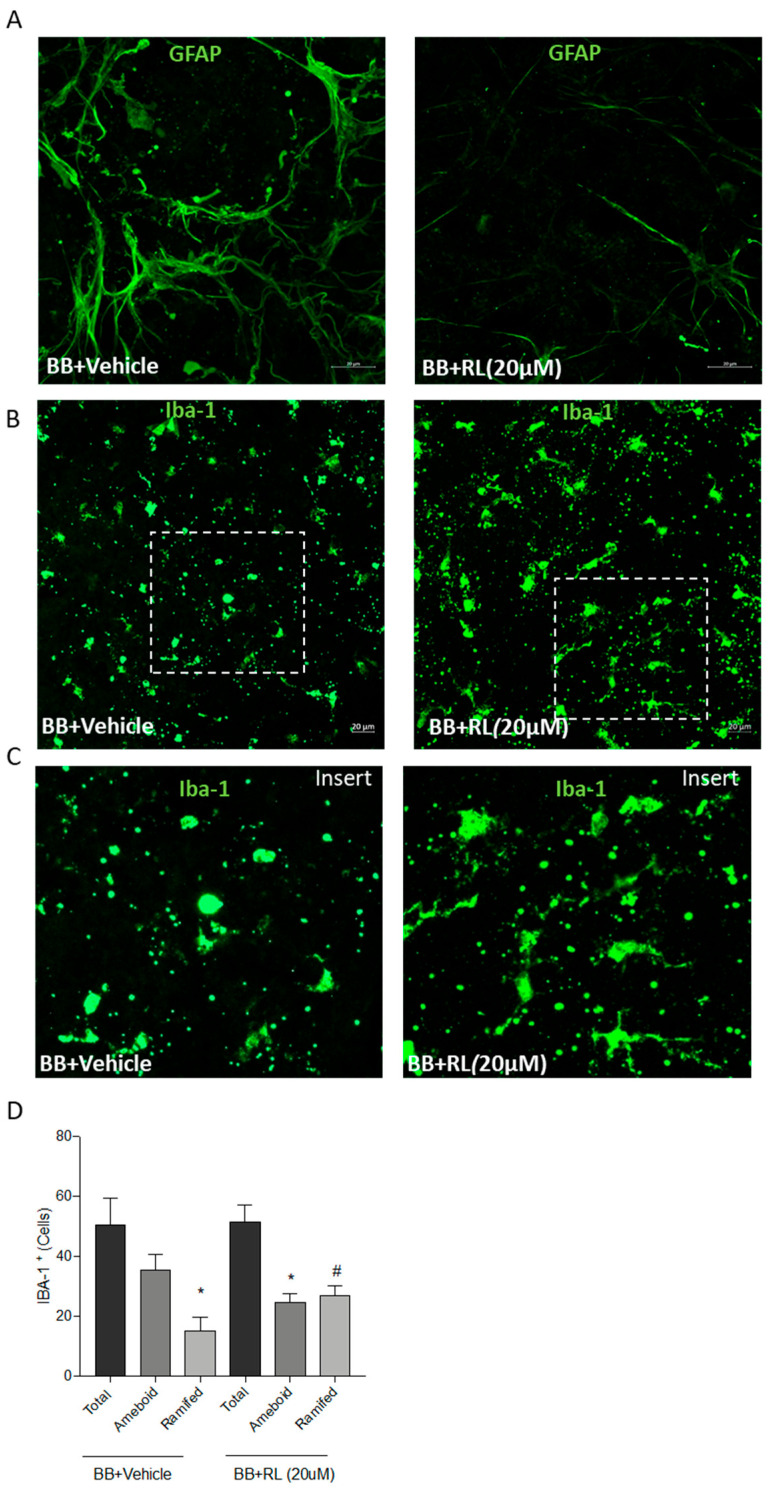
Immunostaining of inflammatory markers in retinal explants from 7-week-old BB rats treated with RL (20 μM) for 24 h. (**A**) GFAP (green) immunostaining for glia cells and (**B**) IBA-1 (green) immunostaining for microglial cells. Scale = 20 μm. Dashed boxes indicate the zoomed-in area displayed in (**C**). (**D**) Quantification of IBA-1^+^ cells (ramified or amoeboid) in retinal explants from BB rats in vehicle condition or RL treatment. * *p* ≤ 0.05 vs. IBA-1^+^ amoeboid vehicle condition, ^#^ *p* ≤ 0.05 vs. IBA-1^+^ ramified vehicle condition (n = 5 retinas per condition). One-way ANOVA followed by Bonferroni *t*-test.

Reactive gliosis is a classical inflammatory event that BB rats exhibit in the early stages of DR [17,19] and can be detected by GFAP immunostaining. As Figure 9A shows, GFAP immunostaining was highly expressed in the retinal explants from 7-week-old BB rats treated with vehicle, but the GFAP immunostaining signal was significantly reduced after treatment with compound RL for 24 h. The optimal dose was determined in a previous dose–response study.

The retinal immune response is mediated by activated microglial cells. The immunostaining for the microglial marker IBA-1 in retinal explants from BB rats showed the major presence of activated amoeboid microglia, associated with an inflammatory status of the retina, which upon treatment with RL suffer a switch in microglial shape towards a non-activated status (ramified microglia) (Figure 9B). The specific phenotypic change induced by treatment with compound RL is shown in a zoomed-in image for each condition (Figure 9C).

Quantification of immunopositive microglial cells in retinal explants from BB rats treated with vehicle (IBA-1^+^) revealed a higher number of amoeboid cells than ramified microglial cells. However, a similar number of amoeboid and ramified microglial cells was detected in retinal explants from BB rats exposed to RL (Figure 9D). This change in the morphology indicates that the anti-inflammatory effect of RL facilitates modulation of microglial response by promoting a phenotype switch from activated (amoeboid) to resting (ramified) microglia.

## 4. Discussion

Diabetic retinopathy, the most common complication of T1DM, is characterized by a progressive degeneration of retinal microvasculature and neurons leading to retinal dysfunction and visual impairment [33]. Most treatments of DR are focused on the late stages of the disease, when the vision has already been seriously damaged, and their use is limited by significant side effects [3]. However, targeting the early processes of DR could lead to better therapeutic responses and improved patient outcomes. In this sense, accumulating evidence indicates that the inflammatory phenomena that occur during DR play a key role in the onset and progression of retinal pathology [34], and, therefore, decreasing early retinal inflammation could be a crucial contribution to DR management. In the search for more effective and specific anti-inflammatory agents, the huge structural diversity of natural products provides great opportunities for the discovery of new compounds capable of regulating retinal inflammatory events. In this study, we have shown that two algal diterpenoids, rugukadiol A (RK) and ruguloptone A (RL), decrease the pro-inflammatory response in retinal innate immune cells (microglia) and in peripheral immune cells (macrophages), with compound RL also able to induce a robust M2 anti-inflammatory response of microglial cells and retinal explants and to promote a phenotypical switch of microglia from activated to resting in an ex vivo DR model.

During T1DM and the progression of DR, the innate immune system becomes activated in response to diabetes-mediated insults [35]. Upon activation, microglial cells, which constitute the retinal immune system, and circulating immune cells, which contribute to the failure of BRB and subsequent infiltration, can adopt two opposite functional states, M1 or M2 (pro- or anti-inflammatory, respectively) [36]. In this study, we examined the immunomodulation that occurs due to the inflammatory response in a DR context and its potential as a therapeutic target. We used LPS as inflammatory stimulus in the Bv.2 microglial cell line and RAW 264.7 macrophage cell line, since the pro-inflammatory response of these cells resembles the in vivo situation during DR in T1DM. We found that pre-treatments with the natural products RK or RL reduce the M1 response induced by LPS in both cell lines, as evidenced by the significant decreases in *Nos2* expression, iNOS level, and mRNA expressions of pro-inflammatory cytokines *Il1b* [27] and *Il6* in comparison with LPS-stimulated and non-treated cells. Further, the *Il6* mRNA inhibitions caused by RL on LPS-stimulated Bv.2 cells and RAW 264.7 cells were similar to those induced by classical anti-inflammatory molecules like dexamethasone. These results are in line with those observed for several natural products, including a few terpenoids, which suppress the M1 pro-inflammatory response of microglia [37] or macrophages [24]. M1 and M2 are the extremes of a dynamic process of polarization, and once the activated immune cell has adopted a phenotype, this still may change in response to new stimuli [12]. Differing from the inhibition of the M1 phenotype, the capability to induce M2 response in microglia has been less frequently identified among natural products [38]. Our results show that compound RL not only decreased M1-related pro-inflammatory factors, but also strongly upregulated the expressions of genes and molecules involved in M2 polarization and M2 phenotype markers, such as arginase-1 and IL-10 [23], and increased the levels of HO-1 protein, which plays a crucial role in the response to microglial cellular damage. As an approach to the treatment of disorders involving immune alterations, increasing attention has focused on the identification of natural compounds featuring a pharmacological profile that induce HO-1 in a cell-specific and cell context-specific manner [39]. Thus, new molecules such as RL, with HO-1 induction capability in microglial cells, represent an interesting approach for therapeutic interventions in DR. On the other hand, the effects of RK on the induction of the M2 response in microglial cells were not as robust regarding the expression of proteins arginase-1 and HO-1.

Despite having similar functions, microglia and macrophages may display different responses depending on the type of stimulus and context [12]. In this study, analysis of the contribution of macrophage cells to the immunomodulation of DR showed that neither RK nor RL treatment enhanced the M2 markers (arginase-1 and HO-1) in RAW 264.7 macrophage cells. These results indicate that, upon treatment with these compounds, microglial and macrophage cells generate an anti-inflammatory response through different signaling pathways. Our results are in line with those recently described for the flavonoid kaempferol [40] and 3-(2,4-dihydroxyphenyl)phthalide [41], which caused stronger M2 responses in microglial cells than in macrophage cells. These differential effects might be due to the compounds’ specific mode of interaction with p38α in each type of immune cell.

On the other hand, HO-1 has been reported to attenuate the activation of NLRP3, a component of the inflammasome that regulates the maturation of pro-inflammatory cytokines [42,43]. NLRP3 orchestrates the responsiveness of immune cells to pro-inflammatory stimuli [44], and an enhanced expression of NLRP3 inflammasome has been described in patients with DR [45,46]. Various natural products of the diterpenoid class have been shown to modulate NLRP3-mediated inflammatory pathways [47], although none of those studies focused on a DR context. In this study, the increased HO-1 levels induced by the treatment of Bv.2 microglial cells with the diterpenoid RL downregulated the canonical pathway of the NLRP3 inflammasome, attenuated the pro-inflammatory effects of several mediators, and promoted the resolution of inflammation. Different mediators, such as the nuclear transcription factor p65-NF*κ*B, are involved in the regulation of the inflammasome complex [48]. The treatment of microglial cells with RL consistently avoided LPS-mediated nuclear translocation of p65-NF*κ*B and decreased NLRP3 protein levels.

P38α-MAPK has long been recognized for its pro-inflammatory functions, and over the years, a large number of structurally diverse compounds, natural or synthetic, have been investigated as p38α-MAPK inhibitors [49]. However, in innate immune cells, p38α-MAPK has also been shown to have anti-inflammatory roles that have been much less explored, including roles mediated by the kinases MSK1/2, which regulate anti-inflammatory genes such as *Il10* [50] and the upstream regulation of HO-1 [51]. It is worth noting that HO-1 and IL-10 mutually reinforce their anti-inflammatory actions. In addition, the induction of IL-10 by HO-1 and the activation of HO-1 by IL-10 both rely on the p38α-MAPK signaling pathway [52,53].

In this study, the treatment of microglial cells with the compound RL alone led to the autophosphorylation of p38α, suggesting a non-classical activation of p38α-MAPK, confirmed by the specific polarization towards M2, which was demonstrated by the induction of the anti-inflammatory mediators IL-10, HO-1, and arginase-1. These effects of RL were similar to those previously observed for several synthetic sp^2^-iminosugar glycolipids [17,19,54] and a 3-arylphthalide [41], suggesting a similar molecular mechanism that involves the autoactivation of p38α upon binding these molecules to a hydrophobic binding site distinct from the classical activation site, and subsequent downstream induction of HO-1 and arginase-1 [17,54].

BB rats, an animal model of autoimmune T1DM, develop DR, which resembles human DR progression [55] and reproduces retinal inflammation with the presence of inflammatory markers and immunomodulation. Previous research has demonstrated that microglia are the main player in DR inflammation, and their modulation by treatment with different molecules results in a delay of DR [19,41]. In this study, the treatment of retinal explants from BB rats in the early stages of DR (at 7 weeks old) with compound RL reduced the M1 pro-inflammatory mediators *Nos2* and *Il1b* and increased M2 anti-inflammatory markers such as *Arg1* and *Hmox1* mRNA expression. These ex vivo results were in full agreement with those observed in vitro upon treatment of LPS-stimulated microglial Bv.2 cells with RL and are also in line with the effects described for other natural compounds [40] and synthetic analogs [19,41].

Glial cells, which provide structural support and are essential for maintaining retinal homeostasis [56], become reactive in the early stages of DR, with morphological and biochemical changes known as reactive gliosis [57]. Specifically, the overexpression of the glial fibrillary acidic protein (GFAP) in Müller glial cells is a hallmark of reactive gliosis and a classical marker of retinal inflammation and tissue damage [57,58]. Moreover, increased levels of GFAP have been reported to happen in the aqueous humor of diabetic patients with DM [59]. In this study, the treatment of retinal explants from BB rats with the compound RL significantly reduced the GFAP immunostaining detected in untreated explants, highlighting the protective effects of RL in a DR environment.

Microglial cells represent the first line of immune defense of the retina. In a healthy retina, resting microglial cells have a ramified appearance and are constantly involved in the surveillance of the surrounding neuronal tissues [60]. However, under different stimuli, microglial cells shift towards an activated status induced by a pro- or anti-inflammatory response. This activation ultimately leads to amoeboid-shaped microglia [60]. Recent ex vivo studies have shown that anti-inflammatory treatments promote a change in the phenotype and function of microglial cells as part of the immune response in DR [18]. In this study, we have found that a decrease in pro-inflammatory signals, together with the induction of M2 response and inflammasome complex modulation under treatment of retinal explants with RL, triggers a shift in activated microglial cells with the amoeboid phenotype into ones with the ramified and resting phenotype.

Previous studies have demonstrated a strong correlation between data obtained from testing active compounds in retinal explants and in vivo models [41], supporting the predictive value of this experimental approach. In line with this evidence, our prior findings reinforce the likelihood that compound RL will exert beneficial effects in vivo. Specifically, RL could be expected to reduce inflammatory parameters associated with diabetic retinopathy, including gliosis, cytokine expression, and the immunomodulation of microglial polarization. Collectively, these data support the potential success of this therapeutic strategy in in vivo settings and warrant further investigation in subsequent stages of pharmacological evaluation.

The pivotal role of inflammation in the development of DR has led to the consideration that modulating the inflammatory processes in the retina could represent an effective therapeutic approach. However, so far the use of anti-inflammatory agents in the treatment of DR is mostly limited to corticosteroids, which are used only in the treatment of advanced DR, with limitations and severe complications [61]. On the other hand, nonsteroidal anti-inflammatory drugs (NSAIDs) have been shown to prevent early DR in animal models, but there are few clinical data, and side effects like cardiotoxicity have also been reported for specific COX-2 inhibitors [62]. In this context, natural products represent an alternative source for the discovery of new, effective, and safe anti-inflammatory agents. Over the last decade, a variety of natural products, most of plant origin, have been investigated for their protective effects on DR [20,63]. In particular, anti-inflammatory effects have been reported for compounds of different classes, including flavonoids, saponins, carotenoids, and terpenoids, which caused decreases in the classical M1-related pro-inflammatory cytokines (IL-1β IL-6, TNF-α) in retinal pigment epithelium cells and in retinal tissue of animal models of DR. In line with these reports, the algal diterpenoids RK and RL suppressed the expression of pro-inflammatory factors in the immune cells involved in DR. Moreover, in this study, we also found that one of these compounds, RL, induced an anti-inflammatory response specifically in the immune retinal cells (microglia), evidenced by the upregulation of IL-10, HO-1, and arginase-1, and these effects were also observed in retinal explants of rats with DR. The anti-diabetic properties of a variety of macroalgal-derived compounds have been investigated in recent years [64], with most studies focusing on the effects on carbohydrate metabolism or insulin signaling and resistance. This is the first account of macroalgae-derived natural products showing activity in the context of DR through modulation of early immune responses and inflammation.

## 5. Conclusions

The identification of natural molecules with immunomodulatory properties provides valuable opportunities for the development of novel therapeutic agents for DR. This study has obtained new insights, through in vitro and ex vivo bioassays, on the anti-inflammatory effects of two marine natural products, rugukadiol A (RK) and ruguloptone A (RL), in the context of DR. In LPS-stimulated microglial and macrophage cells, pre-treatment with RK and RL ameliorated the effects of the phenotype M1 induced by LPS, as reflected by a decrease in the expression of pro-inflammatory mediators. Moreover, in microglial cells, RL not only reduced the pro-inflammatory events and downregulated the inflammasome complex, but also promoted the expression of anti-inflammatory factors characteristic of the M2 phenotype, involving a non-classical p38α-MAPK activation. Likewise, in retinas from 7-week-old T1DM rats, the treatment with RL reduced GFAP immunostaining and increased arginase-1 and HO-1, reflecting anti-inflammatory responses. Taken together, RL has shown to attenuate pro-inflammatory mediators and to promote the resolution of inflammation. This interesting profile of activity opens a path to further explore this class of marine natural products and analogs for early DR management.

## Figures and Tables

**Table 1:** Inhibition rate (%) caused by RK and RL on iNOS and NLRP3 levels and mRNA *Il6* expression in LPS-stimulated Bv.2 and RAW 264.7 cells.

	iNOS Levels		NLRP3 Levels		mRNA *Il6* Expression	
	Bv.2	RAW 264.7	Bv.2	RAW 264.7	Bv.2	RAW 264.7
RK	38.8 ± 1.5	70.7 ± 5.8	65.3 ± 7.0	38.2 ± 2.3	nd	59.1 ± 7.4
RL	57.2 ± 3.2	78.33 ± 1.3	51.2 ± 9.5	64.5 ± 3.6	50.3 ± 11.6	48.5 ± 7.5

nd: No inhibition was detected.

## Data Availability

The original contributions presented in this study are included in the article/Supplementary Materials. Further inquiries can be directed to the corresponding authors.

## Supplementary Materials

The following supporting information can be downloaded at: https://www.mdpi.com/article/10.3390/pharmaceutics18050606/s1, Figure S1: Analysis of markers of M2 response caused by compounds RK and RL in LPS-stimulated RAW 264.7 cells; Figure S2: Ex vivo treatment with RL reduces neuroinflammation in BB rats during DR compared to WT rats; Table S1: List of primers used for mouse and rat.

## Abbreviations

The following abbreviations are used in this manuscript:

DM	diabetes mellitus
DR	diabetic retinopathy
DX	dexamethasone
GFAP	glial fibrillary acidic protein
HO-1	heme oxygenase 1
IBA	ionized calcium-binding adaptor molecule 1
IL-1β	interleukin 1β
IL-6	interleukin 6
LPS	lipopolysaccharide
MAPKs	mitogen-activated protein kinases
NF-κB	nuclear factor kappa-B
NLRP3	nucleotide-binding domain, leucine-rich containing family, pyrin domain-containing-3
NO	nitric oxide
RK	rugukadiol A
RL	ruguloptone A
T1DM	type 1 diabetes mellitus
TGF-β	transforming growth factor β
TNF-α	tumor necrosis factor α
VEGF	vascular endothelial growth factor

## References

[1] Tsalamandris S., Antonopoulos A.S., Oikonomou E., Papamikroulis G.A., Vogiatzi G., Papaioannou S., Deftereos S., Tousoulis D. (2019). The role of inflammation in diabetes: Current concepts and future perspectives. Eur. Cardiol. Rev..

[2] Katsarou A., Gudbjörnsdottir S., Rawshani A., Dabelea D., Bonifacio E., Anderson B.J., Jacobsen L.M., Schatz D.A., Lernmark A. (2017). Type 1 diabetes mellitus. Nat. Rev. Dis. Primers.

[3] Stitt A.W., Curtis T.M., Chen M., Medina R.J., McKay G.J., Jenkins A., Gardiner T.A., Lyons T.J., Hammes H.-P., Simo R. (2016). The progress in understanding and treatment of diabetic retinopathy. Prog. Retin. Eye Res..

[4] Jansson R.W., Hufthammer K.O., Krohn J. (2018). Diabetic retinopathy in type 1 diabetes patients in Western Norway. Acta Ophthalmol..

[5] Madeira C., Lopes M., Laiginhas R., Neves J., Rosas V., Barbosa M., Carvalho D., Falcão-Reis F., Falcão M. (2019). Changing trends in the prevalence of diabetic retinopathy in type 1 diabetes mellitus from 1990 to 2018: A retrospective study in a Portuguese population. Diabetes Res. Clin. Pract..

[6] Simó R., Hernández C. (2022). New insights into treating early and advanced stage diabetic retinopathy. Int. J. Mol. Sci..

[7] Kim T.K., Lee M.S. (2020). Innate immune receptors in type 1 diabetes: The relationship to cell death-associated inflammation. Biochem. Soc. Trans..

[8] O’Leary F., Campbell M. (2023). The blood–retina barrier in health and disease. FEBS J..

[9] Kinuthia U.M., Wolf A., Langmann T. (2020). Microglia and inflammatory responses in diabetic retinopathy. Front. Immunol..

[10] Cherry J.D., Olschowka J., O’Banion M.K. (2014). Neuroinflammation and M2 microglia: The good, the bad and the inflamed. J. Neuroinflamm..

[11] Mosser D.M., Edwards J.P. (2008). Exploring the full spectrum of macrophage activation. Nat. Rev. Immunol..

[12] Funes S.C., Rios M., Escobar-Vera J., Kalergis A.M. (2018). Implications of macrophage polarization in autoimmunity. Immunology.

[13] Strizova Z., Benesova I., Bartolini R., Novysedlak R., Cecrdlova E., Foley L.K., Striz I. (2023). M1/M2 macrophages and their overlaps–myth or reality?. Clin. Sci..

[14] Rath M., Müller I., Kropf P., Closs E.I., Munder M. (2014). Metabolism via Arginase or nitric oxide synthase: Two competing arginine pathways in macrophages. Front. Immunol..

[15] Zhang Y., Zhou A. (2024). Macrophage activation contributes to diabetic retinopathy. J. Mol. Med..

[16] Li X., Yu Z.W., Li H.Y., Yuan Y., Gao X.Y., Kuang H.Y. (2021). Retinal microglia polarization in diabetic retinopathy. Vis. Neurosci..

[17] Alcalde-Estévez E., Arroba A.I., Sánchez-Fernández E.M., Mellet C.O., Fernández J.M.G., Masgrau L., Valverde A.M. (2018). The sp2-iminosugar glycolipid 1-dodecylsulfonyl-5N, 6O-oxomethylidenenojirimycin (DSO2-ONJ) as selective anti-inflammatory agent by modulation of hemeoxygenase-1 in Bv.2 microglial cells and retinal explants. Food Chem. Toxicol..

[18] Arroba A.I., Alcalde-Estevez E., Garcia-Ramirez M., Cazzoni D., de la Villa P., Sanchez-Fernandez E.M., Ortiz-Mellet C., García-Fernández J.M., Hernández C., Simó R. (2016). Modulation of microglia polarization dynamics during diabetic retinopathy in db/db mice. BBA-Mol. Basis Dis..

[19] Cano-Cano F., Alcalde-Estévez E., Gómez-Jaramillo L., Iturregui M., Sánchez-Fernández E.M., García-Fernández J.M., Ortiz-Mellet C., Campos-Caro A., López-Tinoco C., Aguilar-Diosdado M. (2021). Anti-inflammatory (M2) response is induced by a sp2-iminosugar glycolipid sulfoxide in diabetic retinopathy. Front. Immunol..

[20] Zhang J.J., Ni P., Song Y., Gao M.J., Guo X.Y., Zhao B.Q. (2024). Effective protective mechanisms of HO-1 in diabetic complications: A narrative review. Cell Death Discov..

[21] Paine A., Eiz-Vesper B., Blasczyk R., Immenschuh S. (2010). Signaling to heme oxygenase-1 and its anti-inflammatory therapeutic potential. Biochem. Pharmacol..

[22] Campbell N.K., Fitzgerald H.K., Dunne A. (2021). Regulation of inflammation by the antioxidant haem oxygenase 1. Nat. Rev. Immunol..

[23] Kang I.S., Kim R.I., Kim C. (2021). Carbon monoxide regulates macrophage differentiation and polarization toward the M2 phenotype through upregulation of heme oxygenase 1. Cells.

[24] Saqib U., Sarkar S., Suk K., Mohammad O., Baig M.S., Savai R. (2018). Phytochemicals as modulators of M1-M2 macrophages in inflammation. Oncotarget.

[25] Leal M.C., Munro M.H., Blunt J.W., Puga J., Jesus B., Calado R., Rui R., Madeira C. (2013). Biogeography and biodiscovery hotspots of macroalgal marine natural products. Nat. Prod. Rep..

[26] Rocha D.H.A., Pinto D.C.G.A., Silva A.M.S. (2022). Macroalgae specialized metabolites: Evidence for their anti-inflammatory health benefits. Mar. Drugs.

[27] Cuevas B., Arroba A.I., De los Reyes C., Gómez-Jaramillo L., González-Montelongo M.C., Zubía E. (2021). Diterpenoids from the brown alga *Rugulopteryx okamurae* and their anti-inflammatory activity. Mar. Drugs.

[28] Cuevas B., Arroba A.I., de Los Reyes C., Zubía E. (2023). *Rugulopteryx*-derived spatane, secospatane, prenylcubebane and prenylkelsoane diterpenoids as inhibitors of nitric oxide production. Mar. Drugs.

[29] Kristiansen O.P., Mandrup-Poulsen T. (2005). Interleukin-6 and diabetes: The good, the bad, or the indifferent?. Diabetes.

[30] Lang Y., Chu F., Shen D., Zhang W., Zheng C., Zhu J., Cui L. (2018). Role of inflammasomes in neuroimmune and neurodegenerative diseases: A systematic review. Mediat. Inflamm..

[31] Fan J., Xu G., Jiang T., Qin Y. (2012). Pharmacologic induction of heme oxygenase-1 plays a protective role in diabetic retinopathy in rats. Investig. Ophtalmol. Vis. Sci..

[32] Guha M., Mackman N. (2001). LPS induction of gene expression in human monocytes. Cell. Signal..

[33] Du E.J., Sun J.K., Stitt A.W. (2017). Diabetic rethinopathy: Current understanding, mechanisms, and treatment strategies. JCI Insights.

[34] Rübsam A., Parikh S., Fort P.E. (2018). Role of inflammation in diabetic retinopathy. Int. J. Mol. Sci..

[35] Xu H., Chen M. (2017). Diabetic retinopathy and dysregulated innate immunity. Vis. Res..

[36] Yao Y., Li J., Zhou Y., Wang S., Zhang Z., Jiang Q., Li K. (2023). Macrophage/microglia polarization for the treatment of diabetic retinopathy. Front. Endocrinol..

[37] An J., Chen B., Kang X., Zhang R., Guo Y., Zhao J., Yang H. (2020). Neuroprotective effects of natural compounds on LPS-induced inflammatory responses in microglia. Am. J. Transl. Res..

[38] Jin X., Liu M.-Y., Zhang D.-F., Zhong X., Du K., Qian P., Gao H., Wei M.-J. (2019). Natural products as a potential modulator of microglial polarization in neurodegenerative diseases. Pharm. Res..

[39] Funes S.C., Rios M., Fernández-Fierro A., Covián C., Bueno S.M., Riedel C.A., Mackern-Oberti J.P., Kalergis A.M. (2020). Naturally derived heme-oxygenase 1 inducers and their therapeutic application to immune-mediated diseases. Front. Immunol..

[40] Albalawi F.E., Alsharif I., Moawadh M.S., Alkhoshaiban A., Alshehri F.F., Albalawi A.E., Althobaiti N.A., Alharbi Z.M., Almohaimeed H.M. (2024). Immunomodulatory effects of kaempferol on microglial and macrophage cells during the progression of diabetic retinopathy. Int. Immunopharmacol..

[41] Martín-Loro F., Cano-Cano F., Ortega M.J., Cuevas B., Gómez-Jaramillo L., González-Montelongo M.D.C., Freisenhausen J.C., Lara-Barea A., Campos-Caro A., Zubía E. (2024). Arylphthalide delays diabetic retinopathy via immunomodulating the early inflammatory response in an animal model of type 1 diabetes mellitus. Int. J. Mol. Sci..

[42] Ryter S.W. (2019). Heme oxygenase-1/carbon monoxide as modulators of autophagy and inflammation. Arch. Biochem. Biophys..

[43] Ratajczak M.Z., Adamiak M., Ratajczak J., Kucia M. (2021). Heme oxygenase 1 (HO-1) as an inhibitor of trafficking of normal and malignant hematopoietic stem cells–clinical and translational implications. Stem Cell Rev. Rep..

[44] Kelley N., Jeltema D., Duan Y., He Y. (2019). The NLRP3 inflammasome: An overview of mechanisms of activation and regulation. Int. J. Mol. Sci..

[45] Chen H., Zhang X., Liao N., Mi L., Peng Y., Liu B., Wen F. (2018). Enhanced expression of NLRP3 inflammasome-related inflammation in diabetic retinopathy. Investig. Ophtalmol. Vis. Sci..

[46] Kuo C.Y., Maran J.J., Jamieson E.G., Rupenthal I.D., Murphy R., Mugisho O.O. (2022). Characterization of NLRP3 inflammasome activation in the onset of diabetic retinopathy. Int. J. Mol. Sci..

[47] Islam M.T., Bardaweel S.K., Mubarak M.S., Koch W., Gaweł-Beben K., Antosiewicz B., Sharifi-Rad J. (2020). Immunomodulatory effects of diterpenes and their derivatives through NLRP3 inflammasome pathway: A review. Front. Immunol..

[48] Yin Y., Chen F., Wang W., Wang H., Zhang X. (2017). Resolvin D1 inhibits inflammatory response in STZ-induced diabetic retinopathy rats: Possible involvement of NLRP3 inflammasome and NF-kappaB signaling pathway. Mol. Vis..

[49] Wang J., Liu Y., Guo Y., Liu C., Yang Y., Fan X., Yang H., Liu Y., Ma T. (2024). Function and inhibition of P38 MAP kinase signaling: Targeting multiple inflammation diseases. Biochem. Pharmacol..

[50] Reyskens K.M.S.E., Arthur J.S.C. (2016). Emerging roles of the mitogen and stress activated kinases MSK1 and MSK2. Front. Cell Dev. Biol..

[51] Alam J., Cook J.L. (2007). How many transcription factors does it take to turn on the heme oxygenase-1 gene?. Am. J. Respir. Cell Mol. Biol..

[52] Gradziel C.S., Jordan P.A., Jewel D., Dufort F.J., Miller S.J., Chiles T.C., Roberts M.F. (2016). D-3-Deoxy-dioctanoylphosphatidylinositol induces cytotoxicity in human MCF-7 breast cancer cells via a mechanism that involves downregulation of the D-type cyclin-retinoblastoma pathway. Biochim. Biophys. Acta.

[53] Gueder N., Allan G., Telliez M.-S., Hague F., Fernandez J.M., Sanchez-Fernandez E.M., Ortiz-Mellet C., Ahidouch A., Ouadid-Ahidouch H. (2017). sp^2^-Iminosugar α-glucosidase inhibitor 1-*C*-octyl-2-oxa-3-oxocastanospermine specifically affected breast cancer cell migration through Stim1, β1-integrin, and FAK signaling pathways. J. Cell. Physiol..

[54] Padilla-Pérez M.C., Sánchez-Fernández E.M., González-Bakker A., Puerta A., Padrón J.M., Martín-Loro F., Arroba A.I., Fernández J.M.G., Mellet C.O. (2023). Fluoro-labelled sp^2^-iminoglycolipids with immunomodulatory properties. Eur. J. Med. Chem..

[55] Sima A.A.F., Chakrabarti S., Garcia-Salinas R., Basu P.K. (1985). The BB-rat-an authentic model of human diabetic retinopathy. Curr. Eye Res..

[56] Sorrentino F.S., Allkabes M., Salsini G., Bonifazzi C., Perri P. (2016). The importance of glial cells in the homeostasis of the retinal microenvironment and their pivotal role in the course of diabetic retinopathy. Life Sci..

[57] Subirada P.V., Paz M.C., Ridano M.E., Lorenc V.E., Vaglienti M.V., Barcelona P.F., De Luna J., Sánchez M.C. (2018). A journey into the retina: Müller glia commanding survival and death. Eur. J. Neurosci..

[58] Rungger–Brändle E., Dosso A.A., Leuenberger P.M. (2000). Glial reactivity, an early feature of diabetic retinopathy. Investig. Ophthalmol. Vis. Sci..

[59] Vujosevic S., Micera A., Bini S., Berton M., Esposito G., Miden E. (2015). Aqueous humor biomarkers of Müller cell activation in diabetic eyes. Investig. Ophthalmol. Vis. Sci..

[60] Guo L., Choi S., Bikkannavar P., Cordeiro M.F. (2022). Microglia: Key players in retinal ageing and neurodegeneration. Front. Cell. Neurosci..

[61] Ramos H., Hernández C., Simó R., Simó-Servat O. (2023). Inflammation: The link between neural and vascular impairment in the diabetic retina and therapeutic implications. Int. J. Mol. Sci..

[62] Schoenberger S.D., Kim S.J. (2013). Nonsteroidal anti-inflammatory drugs for retinal disease. Int. J. Inflamm..

[63] Zhao Y., Chen Y., Yan N. (2024). The role of natural products in diabetic retinopathy. Biomedicines.

[64] Lauritano C., Iannora A. (2016). Marine organisms with anti-diabetes properties. Mar. Drugs.

