# Supplementary material for: Anti-Inflammatory Effects of the Algal Diterpenoid Ruguloptone A by Modulation of M2 Response in Early Diabetic Retinopathy
Source: Pharmaceutics. 2026 May 15;18(5):606. doi: 10.3390/pharmaceutics18050606 (PMC13210585; doi:10.3390/pharmaceutics18050606)
Supplement: Supplementary file 1 [file pharmaceutics-18-00606-s001.zip › pharmaceutics-4207545-supplementary.pdf]

SUPPLEMENTARY INFORMATION

Anti-inflammatory effects of the algal diterpenoid ruguloptone A by modulation of M2 response in early diabetic retinopathy

Belén Cuevas[1,2], Eva Zubía[2*], Francisco Martín-Loro[1], Ana I. Arroba[1,3*]

[1]Instituto de Investigación e Innovación en Ciencias Biomédicas de la Provincia de Cádiz (INiBICA), Hospital Universitario Puerta del Mar, Cádiz, Spain. belen.cuevagomez@alum.uca.es (B.C), francisco.martin@inibica.es (F. M.-L.)

[2]Departamento de Química Orgánica, Facultad de Ciencias del Mar y Ambientales, Universidad de Cádiz, 11510-Puerto Real (Cádiz), Spain. eva.zubia@uca.es

[3]Departamento de Endocrinología y Nutrición. Hospital Universitario Puerta del Mar, Cádiz, Spain. Ana I. Arroba: ana.arroba@inibica.es (A.I.A.)

*Corresponding authors: Eva Zubía: eva.zubia@uca.es (E.Z.); Ana I. Arroba: ana.arroba@inibica.es (A.I.A.)

Fig. S1 Analysis of markers of M2 response caused by compounds RK and RL in LPS-stimulated RAW 264.7 cells.

Fig. S2 *Ex vivo* treatment with RL reduces neuroinflammation in BB rats during DR compared to WT rats.

Table S1 List of primers used for mouse and rat

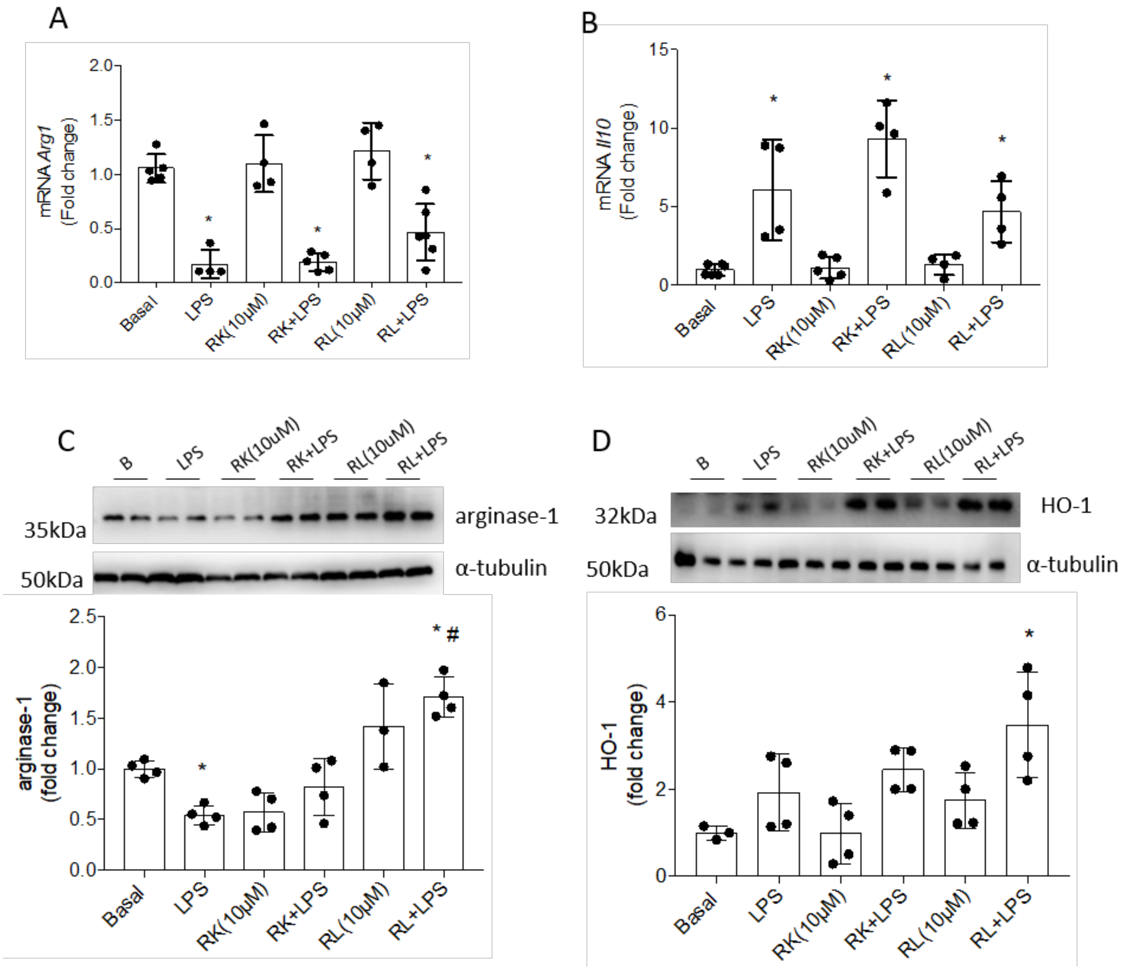

Figure S1 Analysis of markers of M2 response caused by compounds RK and RL in LPS-stimulated RAW 264.7 cells. RAW 264.7 macrophage cells were treated with LPS (200 ng/mL), RK or RL (10 µM), LPS plus RK or LPS plus RL for 24 h. (A) *Arg1* mRNA levels and (B) *Il10* mRNA levels were determined by qRT-PCR. Protein extracts were analyzed by Western blot with antibodies against arginase-1 (C) and HO-1 (D). α-Tubulin was used as a loading control. Representative blots are shown (n=4 independent experiments). Blots were quantified by performing scanning densitometry. The results are mean ± SEM. The fold change relative to the Basal condition is shown. $^*p \leq 0.05$ *vs* Basal, $^{\#}p \leq 0.05$ *vs* LPS, (one-way ANOVA followed by Bonferroni *t-test*)

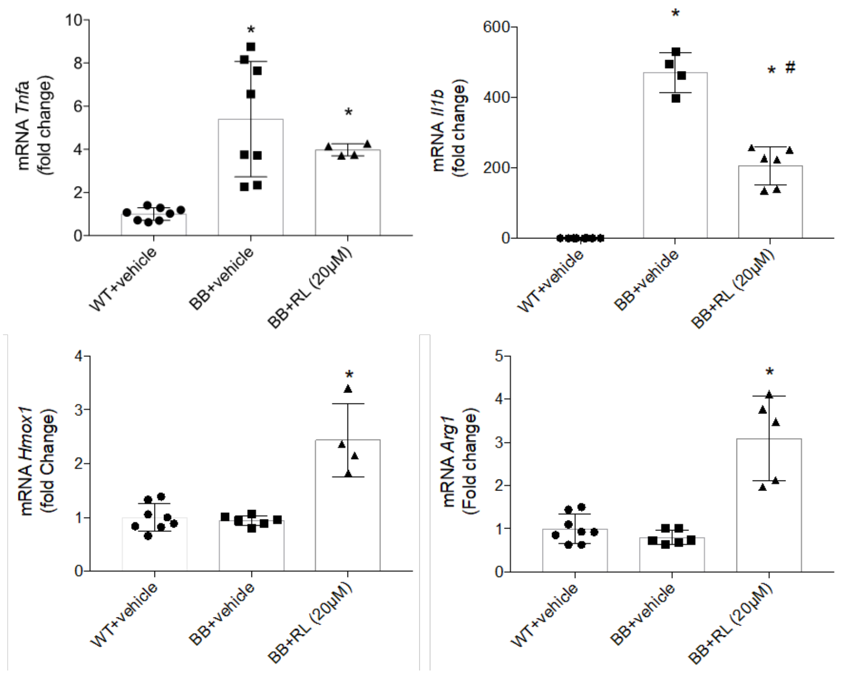

Figure S2 *Ex vivo* treatment with RL reduces neuroinflammation in BB rats during DR compared to WT rats. Retinal explants from WT and BB rats at 7 weeks-old were treated with RL (20 µM) for 24 h. *Il1b* and *Tnfa, Arg1* and *Hmox1* mRNA levels were determined by qRT-PCR. The results are presented as mean ± SEM (n=4-6 retinas per condition). The fold change relative to the WT+vehicle condition is shown. *p ≤ 0.05 *vs* WT+vehicle; # p ≤ 0.05 *vs* BB+vehicle (two-way ANOVA followed by Bonferroni *t-test*)

Table S1 List of primers used for mouse and rat

Gene		*Mus musculus*	*Rattus norvegicus*
Arg1	Forward	CAGAAGAATGGAAGAGTCAG	CCCAGATGTACCAGGATTCTC
	Reverse	CAGATATGCAGGGAGTCACC	TTCCATCACCTTGCCAATC
Actb	Forward	CGCTGTATTCCCCTCCATCG	AGGCCAACCGTGAAAAGATG
	Reverse	CCAGTTGGTAACAATGCCATGT	AGAGCATAGCCCTCGTAGATGG
Hmox1	Forward		TATCGTGCTCGCAATGAACACTCTG
	Reverse		GTTGAGCAGGAAGGCGGTCTTAG
Nos2	Forward		GAGCGCTCTAGTGAAGCAAAG
	Reverse		CTTGCAAGTGAAATCCGATGTGG
Il6	Forward	TACCACTTCACAAGTCGGAGGC	CAGAGGATACCACTCCCAAC
	Reverse	CTGCAAGTGCATCATCGTTGTTC	CAATCAGAATTGCCATTGCAC
Tnfa	Forward		CATCCGTTCTCTACCCAGCC
	Reverse		AATTCTGAGCCCGGAGTTGG
Il1b	Forward		GCAACTGTTCCTGAACTCAACT
	Reverse		ATCTTTTGGGGtCCGTCAACT
	Forward	CGGGAAGACAATAACTGCACCC	GCAGGACTTTAAGGGTTACTTGG
Il10	Reverse	CGGTTAGCAGTATGTTGTCCAGC	GGGGAGAAATCGATGACAGC
